# ALS-linked CCNF variant disrupts motor neuron ubiquitin homeostasis

**DOI:** 10.1093/hmg/ddad063

**Published:** 2023-05-23

**Authors:** Natalie E Farrawell, Monique Bax, Luke McAlary, Jessie McKenna, Simon Maksour, Dzung Do-Ha, Stephanie L Rayner, Ian P Blair, Roger S Chung, Justin J Yerbury, Lezanne Ooi, Darren N Saunders

**Affiliations:** Molecular Horizons and School of Chemistry and Molecular Bioscience, University of Wollongong, Wollongong, New South Wales 2522, Australia; Illawarra Health and Medical Research Institute, Wollongong, New South Wales 2522, Australia; Molecular Horizons and School of Chemistry and Molecular Bioscience, University of Wollongong, Wollongong, New South Wales 2522, Australia; Illawarra Health and Medical Research Institute, Wollongong, New South Wales 2522, Australia; Molecular Horizons and School of Chemistry and Molecular Bioscience, University of Wollongong, Wollongong, New South Wales 2522, Australia; Illawarra Health and Medical Research Institute, Wollongong, New South Wales 2522, Australia; School of Medical Sciences, University of New South Wales, Sydney, New South Wales 2052, Australia; Molecular Horizons and School of Chemistry and Molecular Bioscience, University of Wollongong, Wollongong, New South Wales 2522, Australia; Illawarra Health and Medical Research Institute, Wollongong, New South Wales 2522, Australia; Molecular Horizons and School of Chemistry and Molecular Bioscience, University of Wollongong, Wollongong, New South Wales 2522, Australia; Illawarra Health and Medical Research Institute, Wollongong, New South Wales 2522, Australia; Centre for Motor Neuron Disease Research, Department of Biomedical Sciences, Faculty of Medicine, Health and Human Sciences, Macquarie University, Sydney 2109, New South Wales, Australia; Centre for Motor Neuron Disease Research, Department of Biomedical Sciences, Faculty of Medicine, Health and Human Sciences, Macquarie University, Sydney 2109, New South Wales, Australia; Centre for Motor Neuron Disease Research, Department of Biomedical Sciences, Faculty of Medicine, Health and Human Sciences, Macquarie University, Sydney 2109, New South Wales, Australia; Molecular Horizons and School of Chemistry and Molecular Bioscience, University of Wollongong, Wollongong, New South Wales 2522, Australia; Illawarra Health and Medical Research Institute, Wollongong, New South Wales 2522, Australia; Molecular Horizons and School of Chemistry and Molecular Bioscience, University of Wollongong, Wollongong, New South Wales 2522, Australia; Illawarra Health and Medical Research Institute, Wollongong, New South Wales 2522, Australia; Molecular Horizons and School of Chemistry and Molecular Bioscience, University of Wollongong, Wollongong, New South Wales 2522, Australia; School of Medical Sciences, University of Sydney, Sydney 2006, Australia

## Abstract

Amyotrophic lateral sclerosis (ALS) and frontotemporal dementia (FTD) are fatal neurodegenerative disorders that share pathological features, including the aberrant accumulation of ubiquitinated protein inclusions within motor neurons. Previously, we have shown that the sequestration of ubiquitin (Ub) into inclusions disrupts Ub homeostasis in cells expressing ALS-associated variants superoxide dismutase 1 (SOD1), fused in sarcoma (FUS) and TAR DNA-binding protein 43 (TDP-43). Here, we investigated whether an ALS/FTD-linked pathogenic variant in the *CCNF* gene, encoding the E3 Ub ligase Cyclin F (CCNF), also perturbs Ub homeostasis. The presence of a pathogenic CCNF variant was shown to cause ubiquitin-proteasome system (UPS) dysfunction in induced pluripotent stem cell-derived motor neurons harboring the *CCNF*  ^*S621G*^ mutation. The expression of the CCNF^S621G^ variant was associated with an increased abundance of ubiquitinated proteins and significant changes in the ubiquitination of key UPS components. To further investigate the mechanisms responsible for this UPS dysfunction, we overexpressed CCNF in NSC-34 cells and found that the overexpression of both wild-type (WT) and the pathogenic variant of CCNF (CCNF^S621G^) altered free Ub levels. Furthermore, double mutants designed to decrease the ability of CCNF to form an active E3 Ub ligase complex significantly improved UPS function in cells expressing both CCNF^WT^ and the CCNF^S621G^ variant and were associated with increased levels of free monomeric Ub. Collectively, these results suggest that alterations to the ligase activity of the CCNF complex and the subsequent disruption to Ub homeostasis play an important role in the pathogenesis of *CCNF*-associated ALS/FTD.

## Graphical Abstract

**Graphical Abstract:**
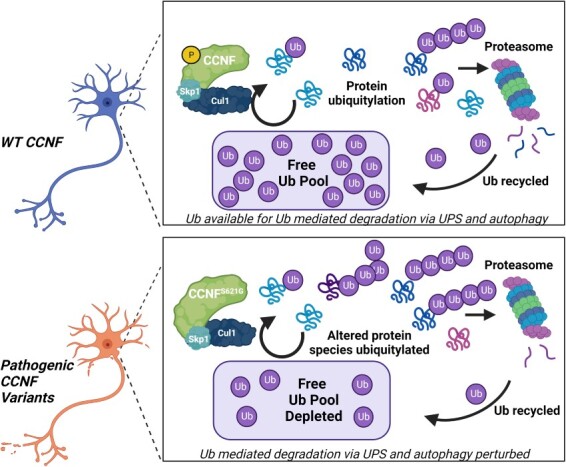

## Introduction

Amyotrophic lateral sclerosis (ALS) is a fatal neurodegenerative disorder characterized by the progressive degeneration of motor neurons in the brain and spinal cord (1). This degeneration manifests as muscle atrophy and subsequent paralysis, typically eventuating in death within 3–5 years of diagnosis. Despite decades of dedicated research, the mechanisms of ALS pathogenesis remain unclear, and in most cases, the cause remains unknown (sporadic ALS). Approximately 10% of cases have an inherited genetic cause (familial ALS), with mutations in over 50 genes currently known to be associated with ALS (2). Up to 15% of ALS patients also develop signs of frontotemporal dementia (FTD) (3) and several genes, including *C9ORF72, TDP-43, VCP, SQSTM1, OPTN, UBQLN2* and *TBK1,* contribute to the etiology of both ALS and FTD (4–6). Strikingly, the large majority of proteins encoded by these genes regulate aspects of intracellular protein degradation, one of three main functional pathways critical for maintaining proteome homeostasis (7). Mutations in *VCP* (8,9), *SQSTM1* (10–12), *UBQLN2* (13), *OPTN* (14,15)*, TBK1* (16,17), *DNAJC7* (18) and *CYLD* (19) have all been associated with ALS/FTD, and all encode components of the ubiquitin-proteasome system (UPS) or autophagic machinery, implicating defective protein degradation in ALS and FTD. Adding to this growing list of protein degradation machinery genes associated with ALS/FTD, pathogenic variants in the *CCNF* gene, encoding the E3 ubiquitin ligase Cyclin F (CCNF), have been identified to cause ALS and FTD (20).

CCNF belongs to the F-box family of proteins, which are characterized by the presence of an F-box motif (21). This motif is crucial to the activity of CCNF as it binds to S-phase kinase-associated protein 1 (SKP1), facilitating interactions with Cullin 1 (CUL1) to form the SKP1-CUL1-F-box E3 ubiquitin ligase complex, mediating the ubiquitination of substrates. The ubiquitination of proteins occurs through a highly ordered three-step process in which ubiquitin (Ub) is first activated by an E1 Ub-activating enzyme and then transferred to an E2 conjugating enzyme, which binds to a specific E3 ligase, such as CCNF, to facilitate Ub attachment to a target substrate (22,23). In the final step, Ub is covalently bound to a lysine residue on a protein substrate, and by cycling through this step multiple times produces distinct poly-Ub chain linkages, which signal a range of essential cellular functions, including protein degradation, signal transduction, endocytosis, transcription and DNA repair (24,25).

In neurons, Ub regulates neuronal growth and survival by contributing to processes, such as synaptic plasticity and neurotransmission (26). Ub exists in a dynamic equilibrium between free Ub and poly-Ub conjugates in cells, with Ub availability controlled through the opposing actions of Ub ligases and deubiquitinating enzymes (DUBs) in addition to the regulation of synthesis and degradation rates (27,28). We have recently shown that the sequestration of Ub into insoluble protein aggregates also alters the cellular availability of Ub (29,30).

The accumulation of ubiquitinated protein inclusions is a hallmark pathology of many neurodegenerative diseases, including ALS and FTD (31). The main pathological constituent of these inclusions varies based upon the disease, whether the disease is sporadic or familial, and the genetics of the familial forms. Although the majority of sporadic ALS and FTD cases have inclusions that are immunoreactive for TDP-43 (32–34), SOD1 and FUS familial ALS cases are associated with the deposition of SOD1 and FUS, respectively, and show no immunoreactivity for TDP-43 (35). This variation is supported by the observation that TDP-43, SOD1 and FUS form distinct inclusions via different pathways in cells (36). Despite these differences, we have recently shown that SOD1, TDP-43 and FUS all co-aggregate with supersaturated proteins (supersaturation is a balance between protein concentration and solubility, i.e. supersaturated proteins have cellular concentrations that exceed their predicted solubility) (37). Consistent with a collapse in the proteostasis capacity of motor neurons, these supersaturated proteins are often associated with cellular quality control machinery, including molecular chaperones and components of the UPS and autophagy pathways (37). In fact, spinal motor neurons have been shown to be particularly susceptible to UPS stress (38), and have a reduced UPS capacity and metastable proteome, compared with oculomotor neurons that are resistant to degeneration in ALS (39,40). Together, this highlights the importance of UPS and Ub homeostasis in the pathogenesis of ALS/FTD.

The functional role of CCNF in the context of ALS/FTD is not yet fully understood. Initial studies have indicated that ALS-associated variants in *CCNF* cause UPS dysfunction (20) and increased K48 polyubiquitination, resulting from changes to the E3 ligase activity of the CCNF complex (41–43). Recently, we have also shown that the expression and aggregation of several ALS-associated proteins lead to UPS dysfunction and perturbed cellular Ub homeostasis (29,30), common features of ALS pathogenesis. Hence, we aimed to examine the effects of pathogenic CCNF on these pathways using induced pluripotent stem cell (iPSC)-derived motor neurons from a symptomatic CCNF^S621G^ ALS patient or healthy control, Ub proteomics and mutants that disrupt the ability of CCNF to form an active Ub ligase complex, coupled with reporters of UPS function and Ub homeostasis. Our findings highlight the importance of Ub homeostasis in ALS.

## Results

### UPS activity is decreased in CCNF^S621G^ motor neurons

**Figure 1:**
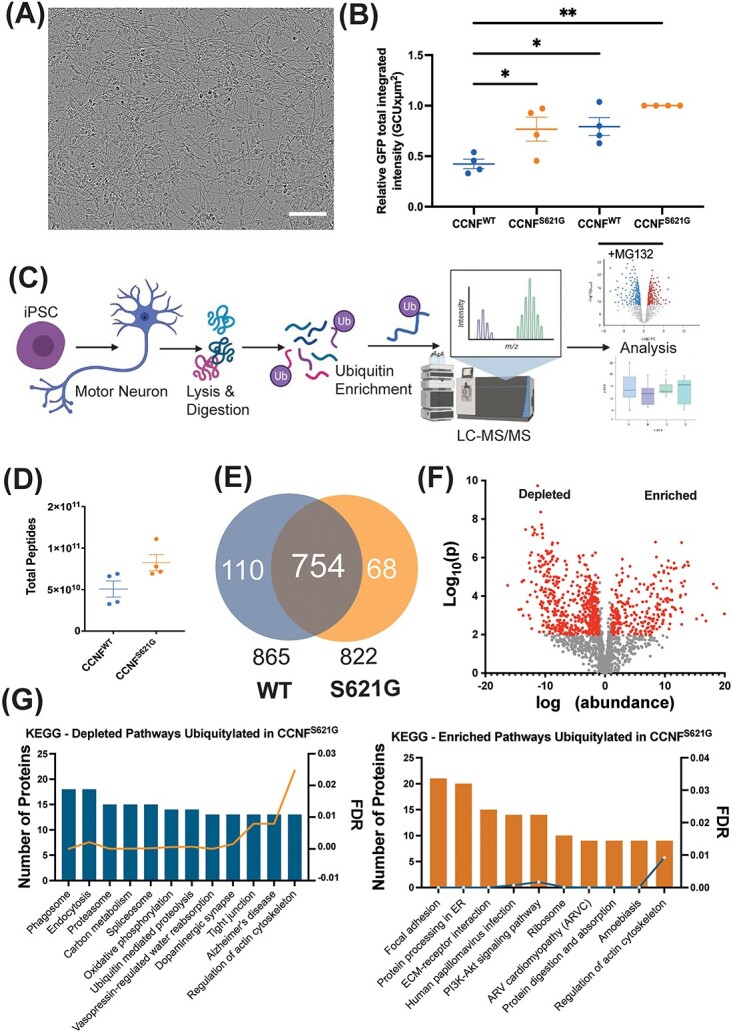
The effect of *CCNF^S621G^* on the ubiquitin-modified proteome of iPSC-derived motor neurons. (**A**) Representative image of iPSC-derived motor neurons differentiated from a symptomatic *CCNF^S621G^* ALS patient and a healthy donor. Cells were compared for UPS efficiency using: (**B**) GFP^u^ degron assay under basal conditions and following proteasomal inhibition with 10 μM MG132. GFP^u^ expression was calculated as GFP total integrated intensity (GCU × μm^2^). Data points show replicates, horizontal bars show mean and error bars denote the standard error of the mean, with each data point representing the average of 9 images across independent differentiations. Significant differences by one-way ANOVA with Tukey’s post hoc multiple comparisons tests: ^*^P < 0.05, ^*^^*^P < 0.01. Data were normalized to MG132-treated *CCNF^S621G^* cells, demonstrating maximal inhibition of the UPS. There was no significant difference between the maximal inhibition of MG132-treated CCNF WT and CCNF^S621G^. (**C**) Schematic of the workflow, showing the derivation of patient-derived motor neuron cultures and ubiquitomics analysis. (**D**) Total peptide counts and (**E**) proteins were identified in the ubiquitome of iPSC-derived motor neurons derived from *CCNF^WT^* and *CCNF^S621G^* donors. (**F**) Volcano plot showing altered expression of individual proteins in the ubiquitome of *CCNF^WT^* and *CCNF^S621G^* expressing motor neurons. (**G**) Functional enrichment analysis of KEGG pathways enriched and depleted in *CCNF^WT^* and *CCNF*^*S621*G^ expressing motor neurons.

Previous work showed that overexpression of CCNF^S621G^ resulted in UPS dysfunction and alterations in the Ub-modified proteome in NSC-34 and Neuro-2A cells (20,42). Here, we sought to examine the effect of the CCNF^S621G^ mutation on the UPS using iPSC-derived motor neurons harboring this mutation instead of an overexpression model. The iPSC lines have previously been characterized (Supplementary Material, Table S1) (44)) and were simultaneously differentiated to motor neurons using a protocol that has been well characterized (38). HB9 and Islet 1 staining were quantified in 75–95% of cells (Supplementary Material, Fig. S1), consistent with our previous findings (38). First, we examined the flux of substrate through the UPS in iPSC-derived motor neurons differentiated from a symptomatic ALS patient heterozygous for the *CCNF^S621G^* variant, and a healthy donor homozygous for *CCNF^WT^* (Fig. 1A, Supplementary Material, Table S1) by monitoring the degradation of a fluorescent substrate containing a CL1 degron sequence (GFP^u^). As GFP^u^ passes through the UPS, including both the ubiquitination and proteasomal degradation steps, it acts as a reporter of UPS efficiency. Our previous work suggests that pathogenic variants of *CCNF* result in the accumulation of GFP^u^ in the absence of proteasome inhibition (20). GFP^u^ expression was calculated as GFP total integrated intensity (GCUxμm^2^) normalized to the CCNF^S621G^ cell line under MG132-induced proteasomal stress, demonstrating maximal inhibition of the UPS. There was no significant difference between the maximal inhibition of MG132-treated CCNF wild-type (WT) and CCNF^S621G^. However, CCNF^WT^ under basal conditions (endogenous stress) had significantly reduced GFP intensity compared with the CCNF^S621G^ under endogenous stress (45% reduction, *P* < 0.05), suggesting impaired GFP^u^ degradation and UPS activity in CCNF^S621G^ motor neurons (Fig. 1B). CCNF^WT^ under endogenous stress also had a significant reduction in relative GFP intensity compared with the CCNF^WT^ and CCNF^S621G^ lines in the presence of 10 μM MG132 (*P <* 0.05 and *P* < 0.01, respectively) (Fig. 1B). There were no significant differences in GFP intensity between CCNF^S621G^ under endogenous stress and CCNF^WT^ or CCNF^S621G^ under proteasomal stress conditions, indicating the UPS system in the CCNF^S621G^ is dysfunctional under basal conditions. Taken together, these findings suggest that the UPS is impaired in CCNF^S261G^ cells, represented by the impaired degradation of GFP^u^, with relative GFP intensity levels comparable to both cell lines under UPS inhibition (Fig. 1B, Supplementary Material, Fig. S2).

Given the biochemical function of CCNF as an E3 Ub ligase, we postulated that this increased load on the UPS was likely because of the previously observed increase in ligase activity (42,43). Examination of the Ub-bound proteome (ubiquitome) using mass spectrometry provides an unbiased and systematic approach to characterize changes in the spectrum of ubiquitinated proteins in cells under different conditions. Hence, to better understand the functional role of CCNF in ALS, we next deployed quantitative proteomics (ubiquitomics) to profile changes in the ubiquitome of iPSC-derived motor neurons from a patient harboring the pathogenic *CCNF^S621G^* variant. Lysates of *CCNF^WT^* and *CCNF^S621G^* iPSC-derived motor neurons were subjected to affinity enrichment and label-free quantitative liquid chromatography–tandem mass spectrometry (LC–MS/MS) to identify differences in the cellular repertoire of Ub-modified proteins (Fig. 1C; Supplementary Material, Table S2; Supplementary Material, Fig. S3). We observed an increased total peptide count following ubiquitin-affinity matrix enrichment of ubiquitinated proteins and ubiquitin-binding proteins (the ubiquitome) from *CCNF^S621G^* cell lysates (Fig. 1D), indicating a greater abundance of ubiquitinated proteins in *CCNF^S621G^* motor neurons compared with *CCNF^WT^* motor neurons. We also observed different ubiquitome repertoires in *CCNF^S621G^* expressing motor neurons compared with *CCNF^WT^* motor neurons (Fig. 1E). Although there were 754 proteins present in common between *CCNF^S621G^* and *CCNF^WT^* motor neurons, we observed 110 proteins present uniquely in the *CCNF^WT^* motor neuron ubiquitome, and 68 proteins uniquely identified in *CCNF^S621G^* motor neurons. This indicates a shift in the spectrum of proteins targeted for ubiquitination by the CCNF*^S621G^* variant.

Quantitative analysis revealed a significant and widespread effect of the *CCNF^S621G^* variant on the motor neuron ubiquitome (Fig. 1F). We observed 256 proteins with significantly enriched abundance in the ubiquitome of CCNF^S621G^ cells, and 545 proteins significantly depleted in CCNF^S621G^ cells, compared with CCNF^WT^. KEGG pathway analysis of these differential ubiquitomes suggests the *CCNF^S621G^* variant perturbs a number of functional pathways in motor neurons. For example, proteins depleted from the ubiquitome of CCNF^S621G^ cells were significantly over-represented in *proteasome* (hsa03050), *spliceosome* (03040) and *Alzheimer’s disease-associated proteins* (hsa05010) KEGG pathways (Fig. 1G, Supplementary Material, Table S3), whereas *focal adhesion* (hsa04510), *protein processing in the endoplasmic reticulum* (hsa04141) and the *ribosome* (hsa03010) were among the top KEGG pathways represented by proteins enriched in the ubiquitome of *CCNF^S621G^* motor neurons. Interestingly, distinct sets of proteins involved in the regulation of the actin cytoskeleton were found to be enhanced or depleted, indicating a significant effect of *CCNF^S621G^* on the motor neuron cytoskeleton.

### Altered UPS regulation in CCNF^S621G^ motor neurons

**Figure 2:**
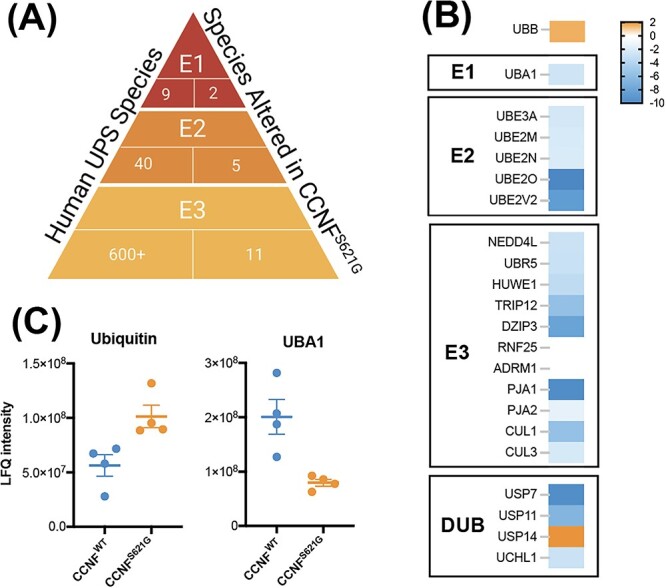
The ubiquitin-proteasome system is modified in *CCNF^S621G^* iPSC-derived motor neurons. (**A**) The number of key enzymes altered at each level of the Ub hierarchy in *CCNF^S621G^* motor neurons. (**B**) Relative abundance of UPS component proteins in the ubiquitome of *CCNF^S621G^* motor neurons compared with *CCNF^WT^* motor neurons. (**C**) Label-free quantitation shows an altered abundance of Ub and UBA1 in the ubiquitome of *CCNF^S621G^* motor neurons.

Consistent with the widespread dysregulation of Ub distribution observed in cells with pathogenic *CCNF* variants (42,43), and extensive cross-talk between components of the Ub conjugation system, we observed significant changes in the abundance of key enzymes at each level in the Ub conjugation hierarchy (E1, E2, E3 and DUBs) in the ubiquitome of *CCNF^S621G^* motor neurons (Fig. 2A). The ubiquitination of the E1 ubiquitin-activating enzyme (UBA1), E2 conjugating enzymes (e.g. UBE2O and UBE2N), E3 ligases (including NEDD4L and RNF25), DUBs (e.g. USP7 and USP11) and subunits of the 19S proteasome cap (e.g. PSMD1, 6, 7, 10, 12 and 13) was modified in *CCNF^S621G^* motor neurons (Fig. 2B). Notably, these components included CUL1 (a component of the SCF E3 ligase complex formed with CCNF), and UBR5, an E3 previously linked to neurodegeneration (45). Of particular interest are decreased abundance of the Ub activator UBA1 in the Ub-modified proteome of *CCNF^S621G^* motor neurons. Pathogenic UBA1 variants result in a rare form of childhood motor neuron disease spinal muscular atrophy (46). Decreased UBA1 signal in the ubiquitome indicates decreased conjugation of UBA1 to Ub and hence decreased UBA1 activity in these cells. Conversely, we observed an increased abundance of Ub in *CCNF^S621G^* motor neurons (Fig. 2C). These widespread effects are consistent with significant disruption or alterations to Ub homeostasis, or reprogramming, of the UPS in *CCNF^S621G^* motor neurons.

### Disruption of CCNF ubiquitin ligase complex formation improves UPS function but does not significantly alter ubiquitin distribution in cells

**Figure 3:**
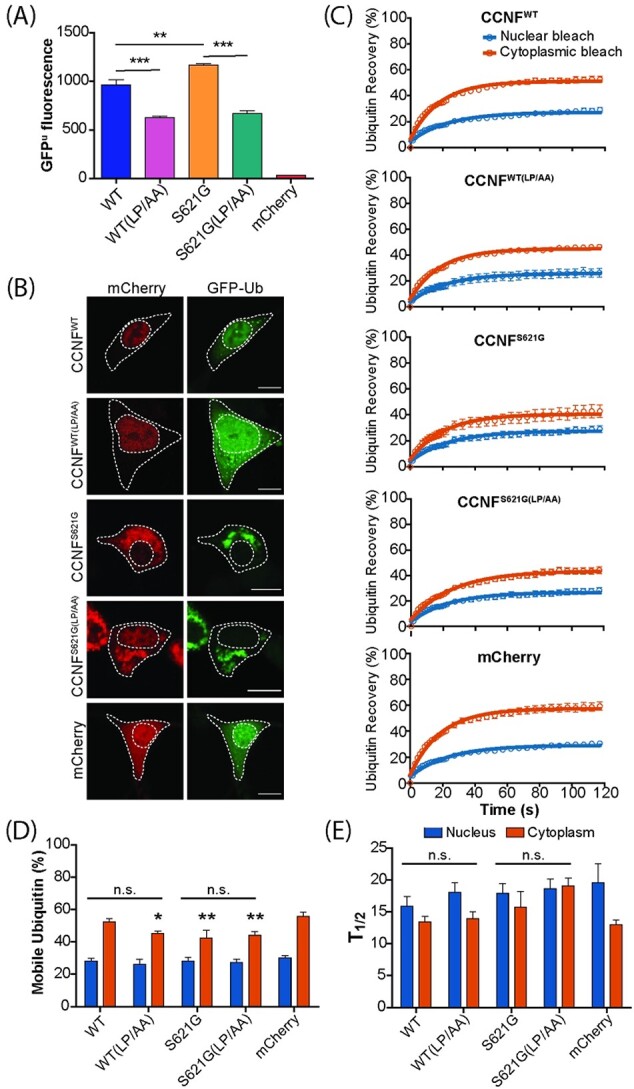
Disrupting CCNF ubiquitin ligase complex formation improves UPS function but does not significantly alter the distribution of ubiquitin in NSC-34 cells. (**A**) UPS activity (GFP^u^ fluorescence) was measured in NSC-34 cells co-transfected with mCherry-CCNF. Data represent mean GFP^u^ fluorescence ± SEM (*n* = 3). One-way ANOVA with a Tukey’s multiple comparisons post-hoc test was used to determine statistical significance (^*^^*^P < 0.01, ^*^^*^^*^P < 0.001). (**B**) Representative confocal images of NSC-34 cells co-transfected with mCherry-CCNF and GFP-Ub. Scale bars represent 10 μm. (**C**) FRAP analysis was performed on NSC-34 cells co-expressing mCherry-CCNF and GFP-Ub by photobleaching a region of interest in either the nucleus or cytoplasm and measuring the recovery of Ub fluorescence over 120 s. (**D**) Quantification of the proportion of mobile Ub in the nucleus and cytoplasm of cells expressing mCherry-CCNF. (**E**) Diffusion rates of GFP-Ub were measured in both the nucleus and cytoplasm of NSC-34 cells co-transfected with mCherry-CCNF. Data shown are means ± SEM combined from 3 independent experiments (*n* ≥ 13). Two-way ANOVA with Bonferroni post-hoc test was used to compare differences between cell populations (n.s. = not significant). Asterisks indicate a significant difference when compared with the mCherry control (^*^P ˂ 0.05, ^*^^*^P ˂ 0.01).

We have previously shown that the expression of ALS-associated CCNF variants in NSC-34 cells caused significant accumulation of the UPS reporter GFP^u^ compared with the expression of CCNF^WT^, indicating that these variants drive UPS dysfunction (20). To further explore the mechanism by which CCNF variants mediate UPS dysfunction, we co-transfected NSC-34 cells with GFP^u^ and mCherry-CCNF constructs. Analysis of GFP^u^ accumulation by flow cytometry revealed that overexpression of both CCNF^WT^ and CCNF^S621G^ led to significantly increased GFP^u^ fluorescence compared with cells expressing mCherry alone (Fig. 3A), suggesting overexpression of CCNF or its increased activity overwhelms the UPS. However, as previously reported (20), GFP^u^ accumulation was significantly higher in cells expressing CCNF^S621G^, compared with cells expressing CCNF^WT^. This effect was independent of CCNF expression levels (Supplementary Material, Fig. S4). Consistent with data that suggests overexpression of CCNF is toxic (47), overexpression of both CCNF^WT^ and CCNF^S621G^ variants led to a substantial accumulation of the UPS reporter when compared with the mCherry alone control. These data are consistent with the overactivity of CCNF, regardless of variant, causing dysfunction and subsequent cell death.

We next investigated the effect of disrupting the ability of CCNF to form an active Ub ligase complex by generating compound mutants containing an F-box mutation (LP/AA), in which the first two amino acids in the F-box domain of CCNF have been mutated to alanine. The LP/AA mutation disrupts the ability of CCNF to form an active Ub ligase complex with SKP1 and CUL1 (48). Accumulation of GFP^u^ was significantly attenuated in cells expressing the compound F-box mutation (LP/AA). Both CCNF^WT^ and CCNF^S621G^ (CCNF^WT(LP/AA)^ and CCNF^S621G(LP/AA)^) expression resulted in significantly less accumulation of GFP^u^ when compared with cells expressing CCNF^WT^ or CCNF^S621G^ alone. These results suggest that the UPS dysfunction associated with CCNF overexpression is at least partially because of the formation of an active E3 Ub ligase complex.

To gain a more detailed understanding of the effects of CCNF mutations on Ub homeostasis, we examined the distribution of Ub in NSC-34 cells over-expressing various mCherry-CCNF variants using fluorescence recovery after photobleaching (FRAP). In cells co-expressing CCNF^WT^, CCNF was predominantly diffuse and localized to the nucleus, whereas GFP-Ub was mostly diffuse throughout the nucleus and cytoplasm, with some small GFP-Ub foci present in the cytoplasm (Fig. 3B). However, in cells expressing the CCNF^S621G^ variants, large cytoplasmic aggregates of both CCNF and Ub formed, which coincided with the depletion of nuclear Ub (Fig. 3B). To determine both the cellular availability of mobile Ub and the rate of Ub diffusion, we bleached an ROI in either the nucleus or cytoplasm of cells co-expressing GFP-Ub and either mCherry-CCNF^WT^, mCherry-CCNF^S621G^, mCherry-CCNF^WT(LP/AA)^, mCherry*-*CCNF^S621G(LP/AA)^ or mCherry only and measured the recovery of GFP-Ub back into this region (Supplementary Material, Fig. S5). In cases where large cytoplasmic aggregates were present, an ROI was selected that contained only soluble material (Supplementary Material, Fig. S5). Recovery dynamics appeared broadly similar between cell populations, with the higher recovery of Ub in the cytoplasm compared with the nucleus (Fig. 3C), consistent with previous reports of greater Ub mobility in this compartment (27). Slightly lower levels of cytoplasmic recovery were observed in cells expressing CCNF^S621G^, in comparison to cells expressing CCNF^WT^ or mCherry alone. However, no obvious differences were observed between cells expressing CCNF with the F-box mutation (CCNF^WT(LP/AA)^ and CCNF^S621G(LP/AA)^) and cells expressing CCNF^WT^ and CCNF^S621G^ without the F-box mutation (Fig. 3C). Analysis of mobile Ub availability revealed that cells expressing CCNF^S621G^, CCNF^WT(LP/AA)^ and CCNF^S621G(LP/AA)^ had significantly lower levels of mobile Ub in the cytoplasm, in comparison to cells expressing mCherry alone (Fig. 3D). This is consistent with the presence of large Ub aggregates in the cytoplasm reducing the availability of mobile Ub (27). No significant differences in the amount of mobile Ub available to cells expressing CCNF with the F-box mutation (CCNF^WT(LP/AA)^ and CCNF^S621G(LP/AA)^) and cells expressing CCNF without the F-box mutation (CCNF^WT^ and CCNF^S621G^; Fig. 3D) were observed, suggesting that disrupting the ability of CCNF to form an active ligase complex does not alter Ub distribution in cells. In addition, no significant differences were observed in the mean half-life of recovery (T_1/2_) between cell populations (Fig. 3E), suggesting that although expression of CCNF^S621G^, CCNF^WT(LP/AA)^ and CCNF^S621G(LP/AA)^ decreased the amount of mobile Ub available to the cell, they did not significantly alter the kinetics of Ub diffusion.

**Figure 4:**
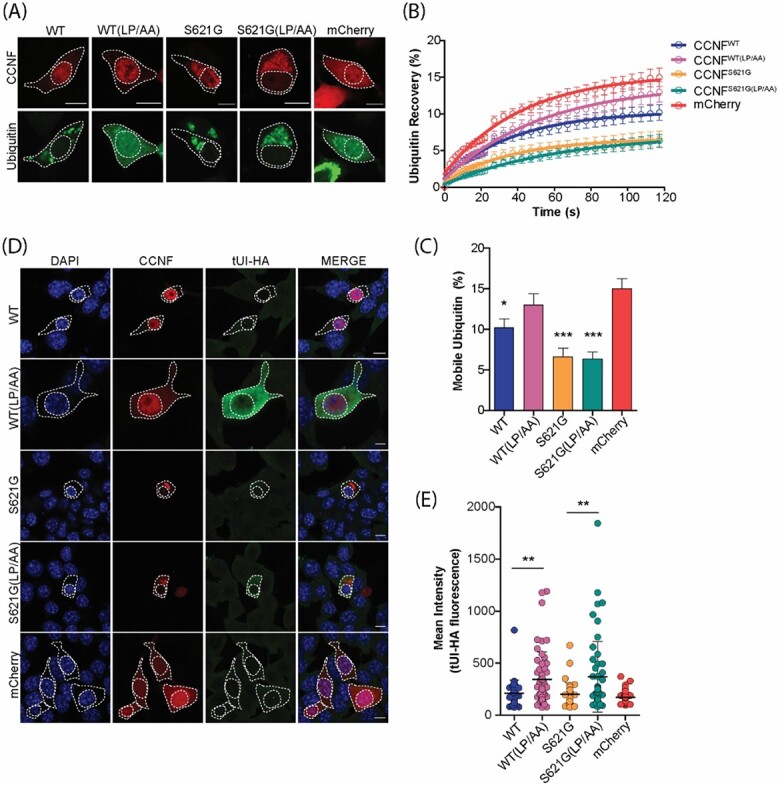
The level of free monomeric ubiquitin is increased in cells with disrupted CCNF ligase complex formation. (**A**) The entire nucleus of NSC-34 cells co-expressing mCherry-CCNF and GFP-Ub was photobleached and (**B**) the recovery of nuclear Ub was monitored as a proportion of cytoplasmic fluorescence for 120 s. Scale bars represent 10 μm. (**C**) The percentage of mobile Ub in the nucleus at the final read was quantified as a proportion of cytoplasmic fluorescence. Data represent mean ± SEM (*n* ≥ 13 combined from 2 independent experiments). One-way ANOVA with a Tukey’s multiple comparisons post-test was used to determine statistical significance compared with mCherry control (^*^P ˂ 0.05, ^*^^*^^*^P ˂ 0.001). (**D**) NSC-34 cells transfected with mCherry-CCNF were fixed, permeabilized and stained for free Ub using the free Ub sensor tUI-HA 48 h post-transfection. Scale bars represent 10 μm. (**E**) Quantification of tUI-HA fluorescence in cells expressing mCherry-CCNF. Data shown are mean ± SD (*n* = 46 CCNF^WT^, *n* = 53 CCNF^WT(LP/AA)^, *n* = 41 CCNF^S621G^, *n* = 45 CCNF^S621G(LP/AA)^, *n* = 146 mCherry). Statistical significance was determined using one-way ANOVA with a Tukey’s multiple comparisons post-test (^*^^*^P ˂ 0.01).

### The pool of free monomeric ubiquitin is increased in cells with disrupted CCNF ligase complex formation

Considering that disrupting the ability of CCNF to form an active ligase complex did not significantly alter the distribution of the total mobile Ub pool when measured by FRAP, we next examined the relative availability of the monomeric form of Ub specifically available to cells using fluorescence recovery after nuclear photobleaching (FRANP). FRANP relies on the nuclear pore acting as a molecular sieve, allowing only the passive diffusion of free monomeric Ub while preventing the diffusion of Ub conjugated in chains (27,29). We bleached the entire nucleus of cells co-expressing GFP-Ub and mCherry-CCNF (Fig. 4A, Supplementary Material, Fig. S6) and quantified the amount of relative monomeric GFP-Ub diffusing through the nuclear pore and into the nucleus (Fig. 4B). Significant decreases in the levels of free monomeric Ub were observed in cells expressing both CCNF^WT^ and CCNF^S621G^ compared with cells expressing mCherry control (Fig. 4C). Interestingly, in some cells, the expression of CCNF^WT^ and CCNF^S621G^ was also associated with the formation of cytosolic Ub aggregates and depletion of nuclear Ub (Fig. 4A). Disrupting the ligase activity of CCNF by introducing the F-box mutation (CCNF^WT(LP/AA)^), restored monomeric Ub availability to a level that was no longer significantly different from the mCherry control. However, there were no significant differences between the levels of free monomeric Ub observed in cells expressing CCNF^S621G^ and CCNF^S621G(LP/AA)^.

The data from the FRANP experiment relies on the co-expression of a Ub-tagged with GFP and does not provide information regarding the endogenously expressed Ub. Next, we analyzed the levels of endogenous free monomeric Ub in CCNF-expressing cells utilizing the free Ub probe tUI-HA. Previous work has demonstrated that tUI-HA has a high affinity and specificity for intracellular free Ub, facilitating the detection and quantification of free Ub in cells (49). Using confocal microscopy, we analyzed tUI-HA fluorescence in NSC-34 cells transiently transfected with mCherry-CCNF or mCherry control (Fig. 4D). Although there were no significant differences in tUI-HA fluorescence between cells expressing CCNF^WT^ and CCNF^S621G^, we observed significant increases in tUI-HA fluorescence in cells expressing F-box mutant CCNF (CCNF^WT(LP/AA)^ and CCNF^S621G(LP/AA)^) compared with CCNF variants that did not carry the F-box mutation (Fig. 4E). This suggests that disruption to the ligase activity of CCNF increases the free monomeric Ub pool in cells.

## Discussion

The recent discovery of ALS and FTD-linked *CCNF* variants further expands a list of genes encoding and regulating components of the UPS implicated in these neurodegenerative conditions. Accordingly, UPS dysfunction is gaining prominence as a key pathological mechanism underlying ALS and FTD. *In vitro* expression of pathogenic *CCNF* variants has been shown to increase the cellular accumulation of ubiquitinated proteins, suggesting that pathogenic effects may be related to disrupted UPS function and Ub homeostasis, but these disease mechanisms are not fully understood. Hence, we performed detailed investigations into the effects of pathogenic *CCNF* variants on cellular Ub homeostasis, the maintenance of which is necessary for neuronal survival. We found a significant accumulation of the UPS reporter GFP^u^ in *CCNF^S612G^* iPSC-derived motor neurons from a symptomatic ALS patient compared with healthy donor iPSC-derived motor neurons. A number of E3 ligases have been identified to facilitate CL1 degron degradation (50), however, it is currently unknown whether CCNF plays a direct role in targeting GFP^u^ for degradation. Ubiquitome analysis indicated an intrinsic UPS dysfunction in the pathogenic *CCNF* variant motor neurons, along with a redistribution of Ub and significant changes in the abundance of key UPS components in the Ub-modified proteome. Disrupting the ability of *CCNF^S612G^* to form an active Ub ligase complex in an overexpression model restored cellular UPS function and increased the pool of free monomeric Ub. Together, these findings suggest that the UPS dysfunction and disruption of Ub homeostasis caused by pathogenic *CCNF* variants result from increased activity of the CCNF ligase complex.

### The UPS is disturbed by pathogenic variant *CCNF*^*S216G*^

UPS impairment underlies many neurodegenerative diseases, including ALS, and overexpression of a number of ALS-associated proteins (including CCNF), can cause UPS dysfunction (20,29,30). Indeed, UPS dysfunction alone has been demonstrated to be sufficient in causing ALS pathology. Motor neuron-specific knockout of the proteasome subunit Rpt3 in mice resulted in the aggregation of ALS-associated proteins, and progressive motor neuron loss and locomotor dysfunction (51). Here, we show for the first time that iPSC-derived motor neurons harboring the *CCNF^S621G^* variant have compromised UPS function, leading to a reduced capacity to degrade a reporter protein targeted to the proteasome. These findings are consistent with previous demonstrations of UPS dysfunction of other ALS-causing genes, whereby GFP^u^ accumulated to a significantly greater extent in ALS TDP-43 patient-derived fibroblasts compared with control fibroblasts (52).

The UPS is a key regulator of cellular processes that are fundamental to maintaining neuronal structure and function, including neurite length and neuronal viability (7,38,53). Our ubiquitomics analysis revealed significantly altered Ub distribution in neurons harboring the pathogenic *CCNF^S621G^* variant, with iPSC-derived motor neurons from an ALS patient containing a 2-fold greater abundance of ubiquitinated proteins relative to WT controls. Similar differences in K48-ubiquitination were reported between Neuro-2A cells expressing CCNF^WT^ and CCNF^S621G^, with proteomic analysis revealing that the ubiquitinated proteins from *CCNF^S621G^*-expressing cells were associated with defective protein degradation via the autophagy pathway (42). Examination of the proteins differentially ubiquitinated in our *CCNF^S621G^*-expressing neurons also highlighted changes to cellular degradation pathways, including Ub-mediated proteolysis and the proteasome.

Furthermore, the abundance of key UPS components in the Ub-modified subset of the proteome, including UBA1, UBR5 and CUL1, were significantly altered in neurons harboring the pathogenic *CCNF^S621G^* variant. Previous ubiquitomics data identified UPS proteins at higher abundance in healthy donor-derived motor neurons, following their differentiation from iPSCs (38). This data highlighted the importance of UPS proteins in healthy motor neuron function (38). In this current study, some of these UPS proteins, including UBA1, E2 proteins UBE2N, UBE2O and the DUB USP7, were found at decreased abundance in *CCNF^S621G^* motor neurons, consistent with significant alterations to Ub homeostasis. UBA1 plays a central role in the UPS, with inhibition of UBA1 causing impaired neurite outgrowth and cell death (38). Dysregulation of UBA1 induces neuromuscular pathology in animal models of spinal muscular atrophy and systemic restoration of UBA1 rescues this pathology (54). Moreover, a number of the largest fold changes in ubiquitinated proteins in the *CCNF^S621G^* motor neurons (Table 1) were in ALS-associated proteins, including DCTN1, ENO2, INA, MAP6 and the more recently associated STMN2, further suggesting a direct role of CCNF in modulating the ALS protein network (55). Collectively, these studies reiterate the importance of the UPS in ALS/FTD pathogenesis. Based on the substantial ubiquitomic disruption in *CCNF^S621G^* motor neurons and the wide-ranging effects of Ub signaling in neurons, we predict that numerous cellular pathways will be affected in *CCNF^S621G^* motor neurons. Additionally, it remains unknown whether there is an increase in misfolded proteins in *CCNF^S621G^* motor neurons. Future studies should identify the impact of altered Ub homeostasis on protein misfolding and *CCNF^S621G^* motor neuron function.

**Table 1:** Top 30-fold changes from CCNF S621G

Downregulated in S621G				Upregulated in S621G			
	Ubiquitylated protein	Fold change (log ratio)	log10pv		Ubiquitylated protein	Fold change (log ratio)	log10pv
1	ENO2	−16.28	4.56	1	MYH3	19.94	3.08
2	ATAT1	−14.39	3.30	2	COL12A1	18.64	4.43
3	GAP43	−14.00	3.32	3	COL14A1	18.11	4.63
4	PRPF38A	−13.88	4.75	4	COL3A1	16.86	2.71
5	NPTX1	−13.69	3.00	5	RRBP1	16.26	3.01
6	MAP6	−13.59	4.82	6	COL1A1	15.30	2.81
7	CTNNA2	−13.28	7.46	7	CKM	13.95	5.59
8	INA	−12.99	3.71	8	COL12A1	13.79	5.77
9	CTNND2	−12.71	3.00	9	COL6A2	13.45	4.09
10	STXBP1	−12.69	5.72	10	KBTBD10	13.29	5.60
11	SCRIB	−12.53	5.44	11	LOXL2	13.29	4.77
12	BTBD17	−12.52	4.06	12	MYLPF	13.05	5.50
13	SEPT3	−12.51	2.83	13	C4A;C4B	13.04	4.51
14	GNG2	−12.49	6.12	14	LEPRE1	12.89	3.17
15	C6orf174	−12.30	4.73	15	P4HA2	12.79	2.53
16	PRKAR2B	−12.27	6.26	16	EML4	12.79	6.78
17	ABAT	−12.24	2.31	17	PDLIM7	12.75	4.69
18	DDR1	−12.14	7.58	18	MEST	12.66	3.36
19	BIRC6	−12.06	7.17	19	MYH8	12.62	4.04
20	ITSN1	−11.82	7.29	20	DES	12.57	3.95
21	MLLT4	−11.77	7.45	21	ENO3	11.87	5.45
22	KIF21A	−11.71	5.49	22	NID1	11.78	3.30
23	NEBL	−11.66	4.24	23	TAGLN	11.57	3.38
24	GSK3B	−11.58	5.29	24	UNC45B	11.53	5.72
25	EDC4	−11.48	4.60	25	MYL1	11.51	5.42
26	STMN2	−11.36	3.95	26	SEPT5	11.22	3.44
27	BCCIP	−11.35	7.20	27	ACTN2	11.20	3.80
28	EPB41L1	−11.26	9.73	28	VWA1	11.13	4.25
29	DCTN1	−11.16	6.15	29	LAMB2	11.08	4.32
30	GNL1	−11.03	5.82	30	COL16A1	10.99	5.47

Since the discovery of pathogenic *CCNF* variants in ALS/FTD, several studies have investigated the disease mechanisms of *CCNF^S621G^,* with a focus on the role of CCNF variants in ligase complex activity. Preliminary studies indicated that *CCNF^S621G^* may cause ALS through UPS dysfunction (20), with more recent studies drawing attention to the prevention of CCNF^S621G^ phosphorylation, resulting in elevated Lys48-ubiquitination activity (41,42). Overexpression of CCNF has been shown to trigger an upregulation of cell death in zebrafish, with transient overexpression of variant CCNF^S621G^ leading to abnormal axonal outgrowth and impaired motor function (47). Together, these results suggest that pathogenic CCNF variants are likely to cause a toxic gain of function. Complementing these findings, we show here that overexpression of CCNF was associated with impaired UPS function, as measured by the increased accumulation of the fluorescent UPS reporter GFP^u^. Interestingly, this impairment was partially rescued by disrupting the ability of CCNF to form an active Ub ligase complex, suggesting that the UPS dysfunction observed with CCNF overexpression results from changes in the ubiquitination activity of the CCNF complex. More specifically, these changes are likely to result from alterations to the ubiquitination kinetics of CCNF, as the F-box variants are still capable of binding substrate but decrease the ubiquitination of substrates (48). Furthermore, it remains possible that mutant CCNF sequesters SCF components in aggregates rendering the SCF complex inactive. Thus, inhibiting the SCF complex even further may aggravate the toxic effect of the mutant CCNF. The potential for the therapeutic application of CCNF will need to be tested in future research.

### Ubiquitin homeostasis is disturbed by pathogenic variant *CCNF*^*S216G*^

The accumulation of ubiquitinated protein inclusions is a major pathological feature of both ALS and FTD (56), and abnormal Ub homeostasis is proposed to play a role in the pathogenesis of ALS. We have previously shown that the expression and aggregation of mutant ALS-associated proteins SOD1, TDP-43 and FUS cause Ub dyshomeostasis and depletion of the free Ub pool in NSC-34 cells (29,30). Here, we show that expression of a pathogenic CCNF variant also perturbs Ub homeostasis, with NSC-34 cells expressing CCNF^S621G^ exhibiting large cytosolic aggregates and decreased levels of mobile Ub (specifically, free monomeric Ub) in comparison to controls. This depletion is consistent with previous findings that the pathogenic CCNF^S621G^ variant causes an accumulation of ubiquitinated proteins through increased activity of the CCNF complex (41,42).

Although disrupting the ability of CCNF to form an active E3 ligase complex did not seem to significantly alter the distribution of the entire mobile Ub pool, disrupting the ligase activity of CCNF did significantly improve the availability of free monomeric Ub to cells. Ub exists in a dynamic equilibrium in cells, and the supply of free Ub must be preserved above threshold levels for the maintenance of the capacity to respond to different cellular stresses (57,58). Depletion of free Ub through modulation of the *UBB* gene results in a neurodegenerative phenotype in mice (59). Strikingly, compensatory expression of Ub through the *UBC* gene is upregulated in neurons from *UBB* deficient mice in an attempt to maintain levels of free Ub and protect against neuronal dysfunction (60). In fact, multiple studies have shown that modulation of the free Ub pool improves neuronal function and survival. Overexpression of Ub rescued the toxicity induced by Ub depletion in yeast (57), and restored free Ub levels in the motor neurons of ataxia mice, improving neurological symptoms (61,62). The UPS, and E3 Ub ligases in particular, may thus offer promise as molecular targets for neurodegenerative drug discovery. A number of small-molecule inhibitors acting on E3 Ub ligase complexes have already been identified as promising drug targets in cancer (63), and considering that free Ub levels were improved by altering the E3 ligase activity of the CCNF complex, modulating this activity may represent a potential therapeutic avenue for the treatment of CCNF-associated ALS. This concept will need to be tested in future research.

In conclusion, we showed that the ALS/FTD-linked variant *CCNF^S621G^* causes UPS dysfunction and disruption of Ub homeostasis in cellular models. These pathologies were partially alleviated by disrupting the ability of CCNF to form an active E3 ligase complex and are consistent with previous findings that suggest that the UPS dysfunction and perturbations to Ub homeostasis caused by *CCNF* result from changes to the E3 ligase activity of the CCNF complex. Together with our observations from other cellular models of ALS, these data highlight the importance of Ub homeostasis in the development of neurodegenerative diseases such as ALS/FTD.

## Materials and Methods

### Plasmids

pmCherry-C1 constructs containing *CCNF^WT^* and *CCNF^S621G^* were generated as described previously (20). *CCNF* F-box variants *CCNF^WT(LP/AA)^* and *CCNF^S621G(LP/AA)^* were obtained from Roger Chung (Macquarie University). GFP–Ub (Addgene plasmid 11 928, deposited by Nico Dantuma) (27) was acquired from Addgene and the GFP^u^ construct was obtained from Ron Kopito (Stanford University) (64).

### Antibodies

The following antibodies were used in this study: rabbit polyclonal anti-HA tag antibody (ab9110, Abcam; 1:1000 dilution), rabbit IgG polyclonal isotype control (ab171870, Abcam), Alexa Fluor 488-conjugated goat anti-rabbit-IgG secondary antibody (A11008, Invitrogen, USA; 1:1000 dilution).

### Expression and purification of tUI-HA

Expression and purification of tUI-HA were carried out as reported by Choi *et al.* (49) with minor alterations, as described in Farrawell *et al.* (30). Briefly, the tUI-HA plasmid was transformed into chemically competent Rosetta BL21(DE3) pLysS cells for protein expression. The dissociated protein was checked for purity via reducing SDS-PAGE before being dialyzed into phosphate-buffered saline (PBS). The concentration of the tUI-HA sensor was determined using UV/VIS spectroscopy (POLARStar Nano, BMG Labtech) at 280 nm using an extinction coefficient of 22 920 M^−1^ cm^−1^ to be 3 mg/ml (65).

### Cell line culture and transfection

Neuroblastoma × spinal cord hybrid NSC-34 cells (66) were maintained in Dulbecco's Modified Eagle's Medium/Ham's Nutrient Mixture F12 (DMEM/F12) supplemented with 10% fetal bovine serum (FBS, Gibco, Australia). Cells were maintained at 37°C in a humidified incubator with 5% atmospheric CO_2_. Cells were plated onto 8-well μslides or multi-well plates 24 h before transfection. Cells were transfected using Lipofectamine 3000 (Invitrogen) or TransIT-X2 transfection reagent (Mirus Bio, USA) according to the manufacturer's instructions. For co-transfections, the amount of DNA was divided equally between constructs.

### iPSC culture and iPSC-derived motor neuron differentiation

All iPSC-based assays were conducted in accordance with the Human Research Ethics Application (HE13/272), in conjunction with a Materials Transfer Agreement (MTA; Ethics HREC/11/CRGH/179). The iPSCs were generated from a healthy 57-year-old male and a 59-year-old male who had symptomatic ALS at the time of collection (44). Cells were cultured under normoxic conditions (37°C, 5% CO_2_). All iPSC lines were generated, characterized and maintained as previously described (44). The iPSC-derived motor neurons were differentiated as per Bax *et al.* (38). Replicates shown are from individual differentiations.

### Ubiquitin proteomics

Ubiquitin proteomic analysis was performed on cell lysates as previously described (38,67). Briefly, 500 μg of total protein was used per sample to immunopurify mono- and poly-ubiquitinated proteins using a specialized ubiquitin affinity matrix (VIVAbind Ubiquitin Kit, VIVA Bioscience, Exeter, UK). After substantial washing to remove residual detergent, beads were digested for 30 min at 27°C, then reduced with 1 mM DTT and left to digest overnight at room temperature (RT) with sequencing-grade trypsin (5 μg/ml, Promega, Madison, WI, USA). Samples were alkylated with 5 mg/ml iodoacetamide and protease digestion terminated with trifluoroacetic acid. Trypsinized eluents were collected after brief centrifugation then purified and desalted using self-packed tips with 6 layers of C18 Empore disks (Pacific Laboratory Products), then dried in a SpeedVac. Samples were then resuspended in 12 μl 5% formic acid, 2% acetonitrile and stored at −80°C. Five microliters of each digested peptide sample were loaded and fractionated along a custom C_18_ column and introduced by nanoelectrospray into an LTQ Orbitrap Velos Pro coupled to an Easy-nLC HPLC (Thermo Fisher). Tandem mass spectrometry data was collected for the ten most abundant ions per scan over a 140 min time gradient and fragmented within the linear ion trap using higher-energy collisional dissociation. The order of data collection was randomized to interchange between biological conditions with bovine serum albumin (BSA) run between each sample to minimize temporal bias. MS/MS data were searched using MaxQuant (v1.2.7.4, Max Planck Institute of Biochemistry, Martinsried, Germany) (68) against the Uniprot human database (release 2016_04). A false discovery rate of 1% was tolerated for protein, peptide, and sites, and one missed cleavage was allowed. Data were filtered for contaminants, reverse hits, proteins only identified by site and number of unique peptides (≥2). Statistical and functional analysis was performed as previously described (38,67).

### UPS activity

UPS activity was measured in NSC-34 cells expressing mCherry-CCNF using the UPS reporter GFP^u^, as described previously (20). Briefly, cells were harvested 48 h post-transfection and GFP^u^ fluorescence was analyzed by flow cytometry on a Becton Dickinson Biosciences LSRFortessa X-20 analytical flow cytometer. UPS activity in iPSC motor neuron precursors was similarly assessed using GFP^u^. On day 21 of differentiation, cells differentiated in a 96-well plate were transfected with Lipofectamine LTX, according to the manufacturer’s protocol. Briefly, liposomes encapsulating a 2:1 ratio of GFP^u^ and mCherry (as a transfection confirmation control) plasmids were added dropwise and incubated with the precursors for 60 h, imaging in-incubator with an Incucyte S3 automated fluorescent microscope (Essen BioScience, USA). Proteasome inhibitor 10 μM MG132 was included in this incubation period to examine the sensitivity of the precursors to UPS stress. Image analysis was performed on cells treated with or without MG132 by filtering cells with mCherry expression and quantifying the GFP expression. GFP^u^ expression was calculated as GFP total integrated intensity (GCU × μm^2^). Data were obtained from the mean of 9 images per replicate, and the mean and standard error of the mean were calculated from four independent replicates.

### Immunofluorescence

Free Ub levels were measured in NSC-34 cells expressing mCherry-CCNF using the high-affinity free Ub sensor tUI-HA, as described previously (30). Briefly, transfected cells in 8-well μslides were fixed with 4% paraformaldehyde before permeabilization with 0.1% Triton X-100 (TX-100) in PBS. Cells were then blocked with blocking buffer (10% FBS, 2% BSA, 0.1% TX-100 in PBS) for 1 h at RT and incubated with the tUI-HA probe diluted 1:400 in blocking buffer for 30 min at RT. Cells were incubated with anti-HA antibody in blocking buffer for 1 h at RT and washed three times with PBS before incubating with Alexa Fluor 488-conjugated secondary antibody in blocking buffer for 1 h at RT. Finally, cells were counterstained with Hoechst 33342 nucleic acid stain (diluted 1:5000 in PBS) for 5 min at RT and imaged on the SP8 confocal microscope.

### Confocal microscopy

FRAP and FRANP experiments were performed on NSC-34 cells 48 h post-transfection using the LASAF FRAP application wizard on the Leica SP5 confocal microscope as described previously (29). Briefly, using the 63× objective and a scan speed of 700 Hz, five pre-bleach images were acquired before the region of interest (ROI) was bleached over five frames with the 488 laser power set to 100%. Recovery of GFP-Ub into the ROI was monitored over 120 s with the laser power set at 20%. For FRAP experiments the ROI was ~ 2 μm in diameter.

To quantify tUI-HA staining, single slice 12-bit images of transfected NSC-34 cells were acquired on the Leica SP8 confocal microscope using the 40× (1.3 numerical aperture) oil immersion objective, one line and frame average with a scan speed of 400 Hz. Images were subsequently processed using ImageJ software (69). Firstly, images from the mCherry channel were thresholded to remove background and the magic wand tool was used to select cells based on fluorescence. Segmentation of cells was performed manually. Regions of interest were applied to the tUI-HA images (green channel) before the mean tUI-HA fluorescence intensity was measured.

### Statistics

All statistical analysis was performed using GraphPad Prism software version 5.00 for Windows unless stated.

*Conflict of Interest statement.* None declared.

## Supplementary Material

HMG-2022-CE-00652-R1_Sup_Material_ddad063

Supplementary_Table_1_ddad063

Supp_Table_2_Intensity_and_LFQs_of_Proteins_Identified_in_CCNF_MNs_ddad063

Supplementary_Table_3_CCNF_KEGG_ddad063

## Data Availability

Data are available upon request.
