# Supplementary material for: ALS-linked CCNF variant disrupts motor neuron ubiquitin homeostasis
Source: Hum Mol Genet. 2023 May 23;32(14):2386–98. doi: 10.1093/hmg/ddad063 (PMC10652331; doi:10.1093/hmg/ddad063)
Supplement: HMG-2022-CE-00652-R1_Sup_Material_ddad063 [file hmg-2022-ce-00652-r1_sup_material_ddad063.docx]

**ALS-linked CCNF variant disrupts motor neuron ubiquitin homeostasis.**

**Natalie E. Farrawell^1, 2, #^, Monique Bax^1, 2, #^, Luke McAlary^1, 2^, Jessie McKenna^3^, Simon Maksour^1, 2,^, Dzung Do-Ha^1, 2,^, Stephanie L. Rayner^4^, Ian P. Blair^4^, Roger S. Chung^4^, Justin J. Yerbury^1, 2^, Lezanne Ooi^1, 2,*^, Darren N. Saunders^1,5^**

^1^Molecular Horizons and School of Chemistry and Molecular Bioscience, University of Wollongong, Northfields Ave, Wollongong, NSW 2522, Australia

^2^Illawarra Health and Medical Research Institute, Wollongong, NSW, Australia

^3^School of Medical Sciences, University of New South Wales, Sydney, NSW 2052, Australia

^4^Centre for Motor Neuron Disease Research, Department of Biomedical Sciences, Faculty of Medicine, Health and Human Sciences, Macquarie University, Sydney, 2109, NSW, Australia

^5^School of Medical Sciences, University of Sydney, Sydney, Australia

# These authors contributed equally.

* Corresponding author:

Prof Lezanne Ooi, Building 32, University of Wollongong, Northfields Ave, Wollongong, NSW 2522, Australia, Tel: +61 2 4221 5865; Email: [lezanne@uow.edu.au](mailto:lezanne@uow.edu.au)

# Supplementary Figures


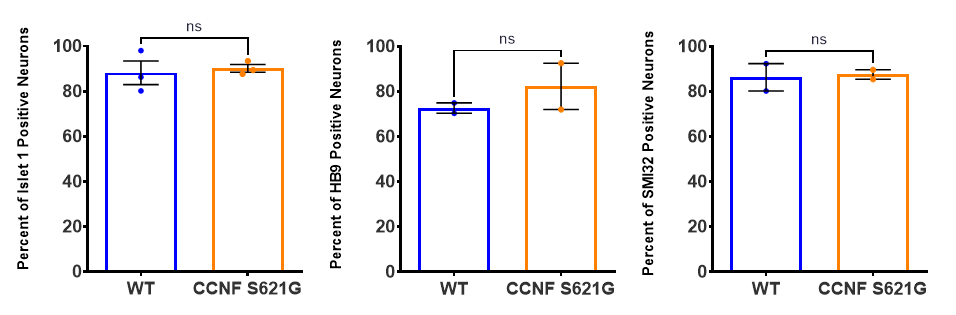


**Supplementary Figure 1:**

The iPSCs (see also Supp. Table 1) were differentiated into motor neurons as per Bax et al., 2019 (38) and Islet 1, HB9, and SMI32 positive cells were quantified by immunocytomchemistry, as described in (38). The results were consistent with our previous data (38), showing (75-95%) motor neurons and no significant difference (ns) between WT and CCNF^S621G^ motor neurons (student’s t-test).

**Supplementary Figure 2:**

The iPSC-derived motor neurons demonstrated a concentration dependent reduction in motor neuron viability with increased MG132 concentration demonstrated concentration dependent effects of MG132 on WT and CCNF^S621G^ motor neurons (n=5 independent differentiations). Viability was assessed by resazurin assay 48 h after MG132 treatment. There were no significant differences between WT and CCNF^S621G^ at any of the concentrations tested.


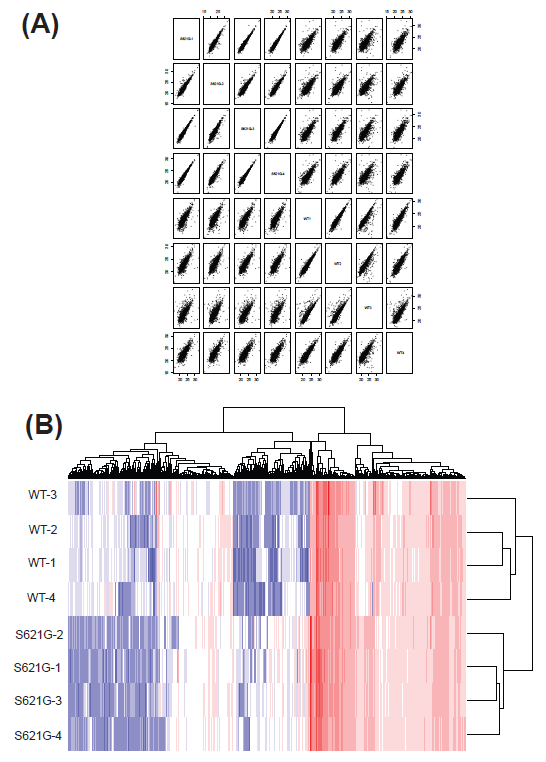


**Supplementary Figure 3:** Scatter plots of the multiple linear regression and (B) Hierarchical clustering showing relationship of individual replicates of *CCNF^WT^* expressing and *CCNF^S621G^*

expressing motor neuron ubiquitomes.


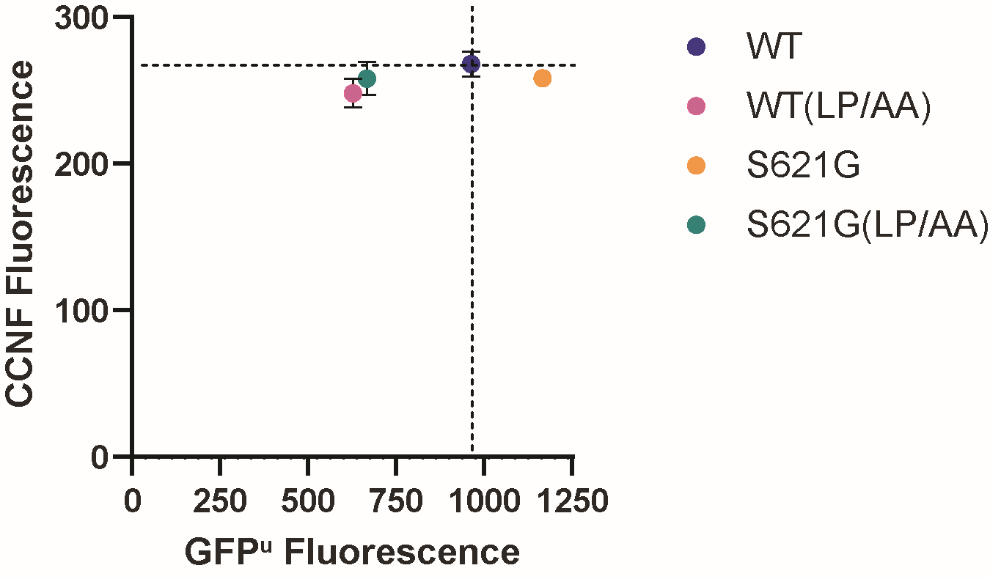


**Supplementary Figure 4:** Levels of GFP^u^ fluorescence were independent of CCNF expression levels. Data represent mean fluorescence ± SD (n=3). Dashed lines indicate WT mean. No statistically significant differences in CCNF expression level were observed, as determined by One-way ANOVA with a Tukey’s multiple comparisons post-test.


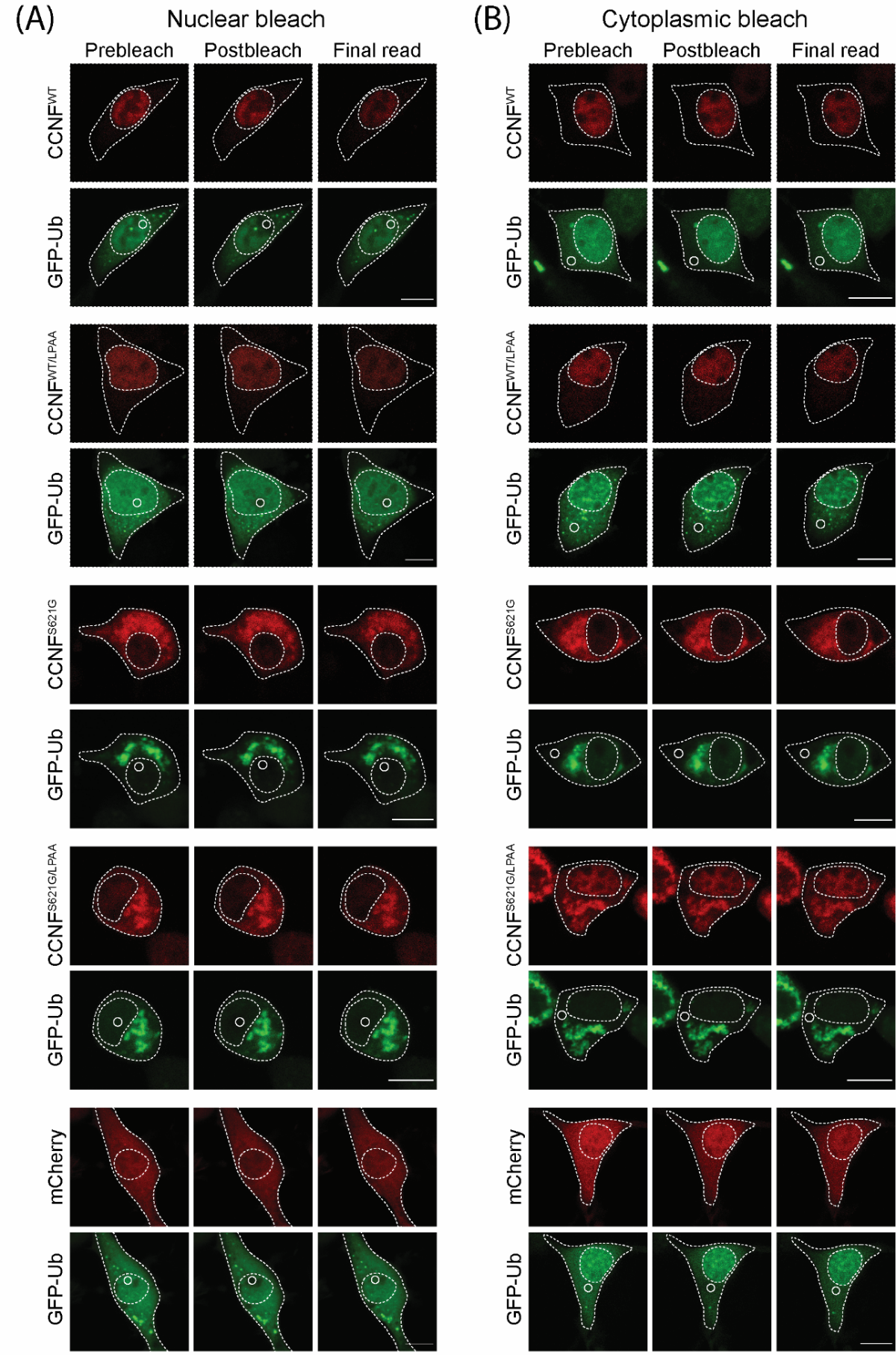


**Supplementary Figure 5:** Representative confocal images of FRAP analysis performed in the nucleus (A) or cytoplasm (B) of NSC-34 cells co-expressing mCherry-CCNF and GFP-Ub. Pre-bleach, post-bleach and recovery endpoint (final read) are shown with the ROI marked by a solid white circle. Scale bars: 10 µm.

**
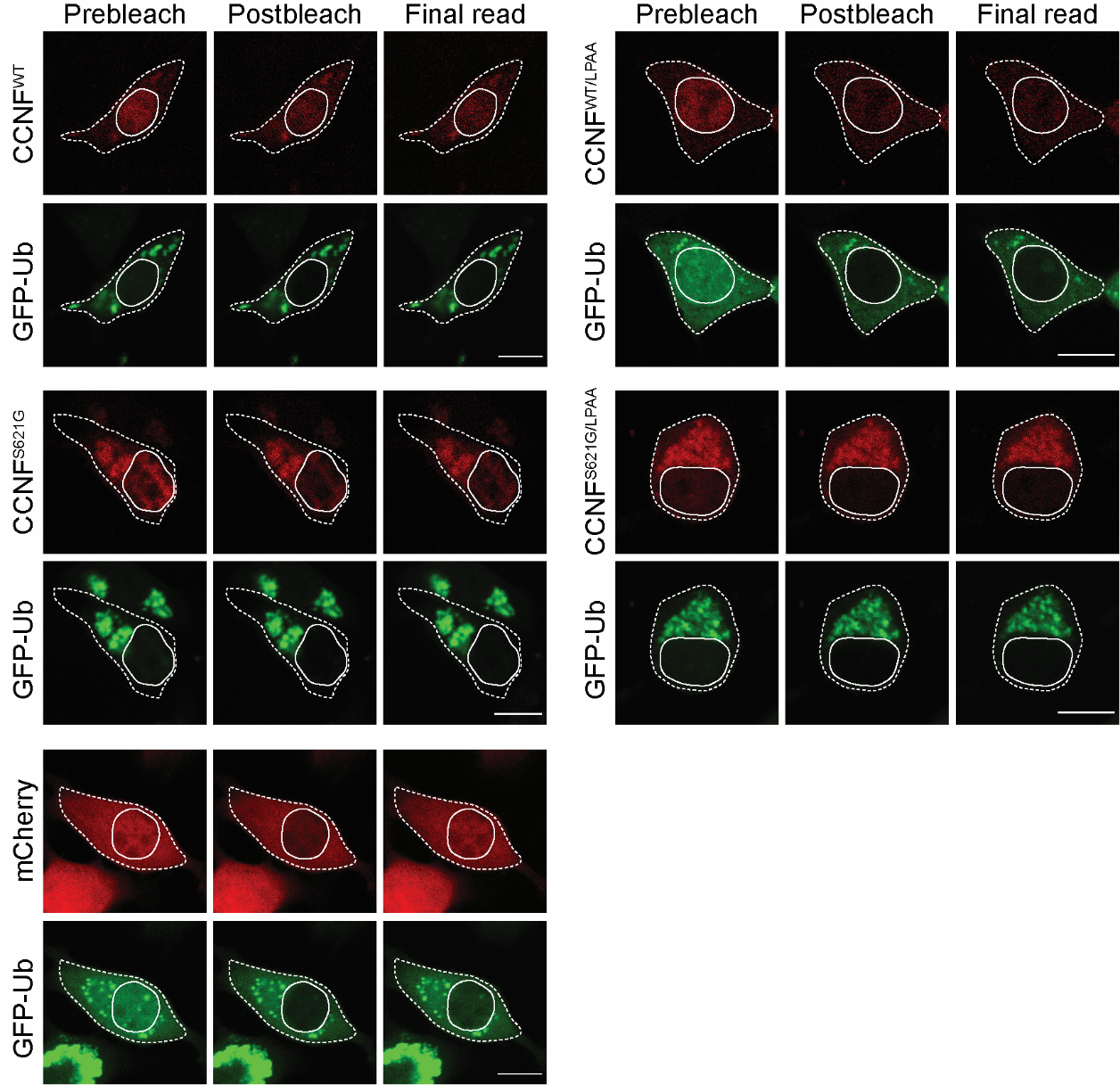
**

**Supplementary Figure 6:** Representative confocal images of FRANP analysis performed on NSC-34 cells co-expressing mCherry-CCNF and GFP-Ub. Pre-bleach, post-bleach and recovery endpoint (final read) are shown with the ROI (nucleus) marked by a solid white line. Scale bars: 10 µm.
