# Supplementary material for: ALS-linked CCNF variant disrupts motor neuron ubiquitin homeostasis
Source: Hum Mol Genet. 2023 May 23;32(14):2386–98. doi: 10.1093/hmg/ddad063 (PMC10652331; doi:10.1093/hmg/ddad063)
Supplement: Supplementary_Table_1_ddad063 [file supplementary_table_1_ddad063.docx]

**Supplementary Table 1**

| Name | Sex | Age | Status | Reference |
| --- | --- | --- | --- | --- |
| CCNFWT | M | 57 | Healthy | (1) |
| CCNFS621G | M | 59 | ALS | (2) |

**References**

1. Chung, H.C., Lin, R.C., Logan, G.J., Alexander, I.E., Sachdev, P.S., Sidhu, K.S. (2012) Human induced pluripotent stem cells derived under feeder-free conditions display unique cell cycle and DNA replication gene profiles. *Stem Cells Dev.* **21** 206-16.
2. Bax, M., Balez, R., Munoz, S.S., Do-Ha, D., Stevens, C.H., Berg, T., Cabral-da-Silva, M.C., Engel, M., Nicholson, G., Yang, S. *et al.* (2019) Generation and characterization of a human induced pluripotent stem cell line UOWi005-A from dermal fibroblasts derived from a CCNFS621G familial amyotrophic lateral sclerosis patient using mRNA reprogramming. *Stem Cell Res.* **40** 101530.
